# Alleviating Costs for Critical Eye Specialty Services (ACCESS): a prospective cohort analysis of a cost-coverage program at a public safety-net hospital

**DOI:** 10.1016/j.lana.2026.101450

**Published:** 2026-03-13

**Authors:** Ryan A. Morton, Irene J. Pak, Alice S. Tang, Deborah Chesky, Hemal K. Kanzaria, Madeline Yung, Tyson N. Kim

**Affiliations:** a Department of Ophthalmology, University of California-San Francisco, San Francisco, CA, USA; b All May See Foundation, San Francisco, CA, USA; c Department of Emergency Medicine, University of California-San Francisco, San Francisco, CA, USA

**Keywords:** Health equity, Insurance coverage, Vision care, Corneal ectasia, Scleral lens

## Abstract

In the United States, gaps in public insurance coverage and high out-of-pocket costs for specialty ophthalmic services contribute substantially to preventable vision loss and disability among underserved populations. We describe the structure, implementation, and early outcomes of ACCESS (Alleviating Costs for Critical Eye Specialty Services), a hospital-based cost-coverage program established at an urban safety-net hospital to address these financial barriers. ACCESS delivers essential vision-restorative treatments frequently excluded from insurance coverage at no cost through hospital support and philanthropic funding. Prospective clinical and demographic data demonstrated marked reductions in visual disability among treated patients and substantial downstream economic benefit relative to program costs. These findings demonstrate that targeted cost-coverage programs embedded within safety-net systems can reduce disparities in vision care, while providing actionable evidence to inform hospital policy development and support broader expansion of insurance coverage for high-impact ophthalmic specialty services.

## Background

Limited health insurance coverage is widely recognized as a driver of disparities in healthcare access and outcomes.^1^, ^2^, ^3^ The consequences of underinsurance are profound, including higher rates of mortality and disability, reduced earnings due to illness, and increased incidence of advanced-stage illnesses.^4^^,^^5^ In eye care, inadequate coverage delays or prevents effective treatment of sight-threatening conditions. This leaves vulnerable, under-resourced patients at greater risk for visual impairment and associated functional limitations that interfere with daily living, which could otherwise have been avoided.^2^^,^^3^^,^^5^^,^^6^ The economic impacts are substantial; estimates on the national burden of vision loss in 2022 exceeded $130 billion United States dollars (USD), with California accounting for $13.5 billion USD of those costs.^7^ Many cases are preventable or even reversible with targeted, cost-effective interventions that can improve individual function and enable fuller participation in society.

Institutional solutions to address preventable vision loss in underserved populations remain rare and fragmented. Zuckerberg San Francisco General Hospital and Trauma Center (ZSFG), the primary safety-net hospital for San Francisco and northern San Mateo counties, serves a patient population in which a substantial proportion are uninsured or underinsured. In 2023, 24.5% of patients at ZSFG were found to be uninsured, and 60% were dependent on public insurance programs for low-income individuals that frequently exclude specialty ophthalmic care.^8^

While some health plans offer optional vision insurance, the additional cost is often prohibitive for patients with limited financial means.^6^^,^^9^ Even when obtained, vision insurance typically does not fully cover medically necessary treatments, such as rigid gas-permeable (RGP) lenses and scleral lenses for corneal diseases, and compounded eye drop medications. RGP and scleral lenses address conditions such as corneal ectasia, highly irregular astigmatism, and corneal scarring, for which eyeglasses or conventional contact lenses are ineffective.^10^^,^^11^ Scleral lenses also provide continual hydration and protection of the ocular surface, controlling pain and preventing sight-threatening complications such as corneal thinning and perforation in patients with severe ocular surface diseases.^2^^,^^11^^,^^12^ Compounded eye drops are likewise critical to treat complex and often rare ocular diseases for which commercially manufactured medications are unavailable. Fortified topical antibiotics can be vision-saving in infectious keratitis.^13^ Topical chemotherapeutic agents are often first-line or adjuncts in the management of ocular surface squamous neoplasia. Autologous serum tears ameliorate severe or refractive ocular surface disease, including keratoconjunctivitis sicca and limbal stem-cell deficiency.^4^ However, these medications require specialized preparation at a compounding pharmacy, are not available for outpatient dispensing at ZSFG, and are rarely covered by insurance.

Such gaps in coverage and accessibility create structural barriers that delay diagnosis and treatment, increasing the risk of disease progression, emergency department utilization, and permanent visual disability.^2^^,^^12^ As a result, underinsured patients face untenable choices: inpatient admission solely for administration of compounded drops, prescriptions for compounded medications they cannot afford to fill, or recommendations for unaffordable contact lenses. These barriers are further exacerbated for patients who are non-English speaking, unhoused, undocumented, or living below the poverty line—demographics that characterize much of ZSFG's patient population.^14^

In line with priorities articulated by global commission reports on eye health, programs that prioritize vulnerable groups, identify cost-effective interventions that respond to community needs, and reduce out-of-pocket costs for these targeted interventions are essential to closing gaps in vision-health equity.^15^^,^^16^ These principles are not limited to low-resource countries; they are highly relevant to safety-net settings in high-income regions where underinsurance and systemic barriers persist. To address these barriers, ophthalmologists and optometrists at ZSFG—together with medical students and volunteers from the University of California, San Francisco (UCSF)—established the ACCESS (Alleviating Costs for Critical Eye Specialty Services) program. ACCESS provides essential, medically necessary ophthalmic specialty services at no cost to patients whose insurance coverage is inadequate or nonexistent. In this report, we describe the program's design, implementation, and outcomes, and offer recommendations for long-term sustainability.

## Search strategy and selection criteria

We performed a structured search to identify academic literature, policy documents, and program descriptions relevant to vision health disparities and coverage gaps for vision-restorative interventions in US safety-net settings. Searches were performed across PubMed, Google Scholar, and Medline for articles published from Jan 1, 1999, to Aug 31, 2025, using combinations of terms including “vision health disparities”, “safety-net hospital”, “uninsured”, “underinsured”, “Medicaid”, “Medi-Cal”, “Medicare”, “insurance coverage eye care”, “scleral lenses”, “rigid gas-permeable lenses”, “keratoconus”, “corneal ectasia”, “corneal opacity”, “compounded ophthalmic medications”, “economic burden”, and “cost-coverage program”. We also reviewed publicly available population-level data sources (e.g., the US Census Bureau's American Community Surveys) to contextualize sociodemographic patterns relevant to safety-net populations.

To explore policy activity and implementation details, we retrieved primary legislative and regulatory materials from official government sources and legislative archives such as LegiScan, including Medi-Cal policy and related DHCS materials on school-based mobile optometric services, and federal proposals to expand Medicare vision benefits. Additionally, we reviewed global and national context documents on eye health and access to care, as well as established health equity initiatives and volunteer-based mobile/outreach programs from their official websites. We prioritized peer-reviewed studies, government documents, and program materials that directly addressed disparities, insurance/coverage barriers, implementation models, and economic burden. We excluded non-peer-reviewed studies and commentaries lacking primary supporting documentation.

## Methods

### Establishment of the ACCESS program

At ZSFG, limitations in insurance coverage have often posed significant challenges for low-income patients accessing critical eye care services.^17^ In response, ACCESS was established to deliver high-impact interventions that are feasible within resource constraints and can meaningfully improve vision and quality of life. Targeted services include scleral, RGP, and soft contact lenses; prescription glasses; and compounded eye drops such as autologous serum tears, fortified antibiotics, topical chemotherapy, and low-dose atropine. Eligible patients are identified by ophthalmologists based on financial need and clinical indications, such as corneal ectasia, corneal opacity, dry eye conditions, refractive error of non-corneal origin, and other ocular pathology. Those requiring refractive correction are referred to on-site optometrists or contracted off-site providers for evaluation and dispensing of appropriate contact lenses or eyeglasses at no out-of-pocket cost to the patient. Compounded medications are prescribed by ZSFG Ophthalmology Clinic staff and shipped directly to the patient from a contracted compounding pharmacy for outpatient use. Available medications for patients are provided in Supplementary Table S3.

**Fig. 1:**
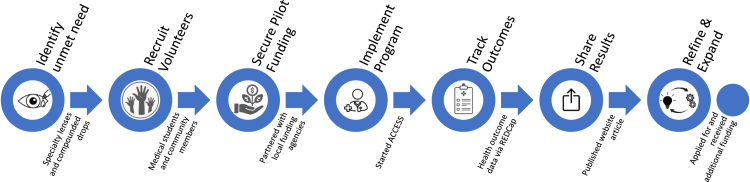
**Implementation timeline of the ACCESS program.** Schematic outlining the inception and development of the ACCESS program at ZSFG. Key steps and milestones include identifying the unmet need for specialty lenses and compounded eye drops, recruiting medical students and community volunteers, securing initial pilot funding, successfully implementing the program, tracking patient health outcomes, publishing results online, and acquiring renewal funding to continue improving and expanding the program.

As illustrated in Fig. 1, the ACCESS program was launched in response to a clear and pressing need for coverage of key treatments that would significantly improve patient outcomes and quality of life. The program has developed through the following steps: (1) clinician identifies medical necessity and coverage gap; (2) volunteers are recruited to help address the problem; (3) funding is secured through institutional grant opportunities; (4) team implements program and begins enrolling patients based on clinical indication and financial need. Operationally, once a patient is identified, the workflow proceeds through referral and eligibility confirmation, coordination of the indicated service (e.g., specialty contact lens evaluation/fitting or compounded medication fulfillment), delivery of the intervention, and follow-up to document outcomes and completion status. (5) These outcomes are then tracked and analyzed to contextualize program impact; (6) program results are shared with funding; and (7) renewal and expansion funding is pursued to sustain and improve the program over time.

In 2021, initial support was secured with stakeholder groups committed to improving community health, including the ZSFG Department of Care Coordination. We analyzed outcomes from the program's first year and leveraged them to obtain additional grants, ensuring the program's continuity. UCSF medical students, faculty, and staff at the ZSFG Ophthalmology and Optometry Clinic collaborated to secure funding from the SFGH Foundation, City and County of San Francisco, Department of Public Health, and external philanthropic organizations. A key partnership was also established with That Man May See, Inc. doing business as All May See Foundation (AMSF), a 501(c) (3) public charity and UCSF affiliate. AMSF provides philanthropic support for UCSF Department of Ophthalmology and Francis I. Proctor Foundation's research, education, patient care, and outreach efforts. AMSF offered fundraising support and provided valuable publicity for the ACCESS program through its website and *Vision* magazine.^18^^,^^19^ Sustainable alignment among ZSFG, UCSF, AMSF, and grant-making and philanthropic partners has been critical to the program's success, while the commitment of motivated students, dedicated volunteers, AMSF leadership, and engaged faculty and clinic staff continues to drive its vitality and growth.

### Data collection and analysis

Data on treatments provided or referred through the ACCESS program, along with vision outcomes and patient testimonials, were prospectively recorded in a REDCap database (Research Electronic Data Capture; Vanderbilt University, Nashville, TN, USA), a secure, web-based platform for building and managing research data. Data entry was performed by clinic staff and providers throughout the program timeline. For analysis, REDCap data were extracted and supplemented with manual chart review to confirm diagnostic indications and collect additional variables, including patient demographics, insurance status, and uncorrected visual acuity (Snellen) before and after ACCESS treatment, when available. We summarized participant flow and the availability of key variables using a STROBE (Strengthening the Reporting of Observational Studies in Epidemiology) diagram, which details enrollment, treatment completion status, and which outcome were available for analysis (Supplementary Figure S1). Demographic variables included age, documented sex, and self-identified race and ethnicity. Because race and ethnicity can be influenced by sociopolitical variables and reflect lived experiences, we applied the UCSF Health *Derived Race and Ethnicity* classification to standardize stratification and reflect local populations, including Latine and multiracial/ethnicity groups.^20^ Additionally, race and ethnicity were variably documented in the electronic health records and not consistently available as separate structured fields; therefore, we report this derived measure as a summary variable rather than separate race and ethnicity.

Diagnostic indications were categorized into five groups: refractive error of non-corneal origin, corneal opacity, dry eye, corneal ectasia, and other ocular pathology (Supplementary Table S1). Patient zip codes were linked to median household income and the percentile of insured adults using the American Community Survey (ACS)^21^ and to vision difficulty percentiles using the Healthy Places Index.^22^ We also performed this analysis for median income at the census tract level; however, due to the small number of patients we avoid showing their geographic distribution at the census tract level. Instead, we provide the broad geographic distribution of single household median income in the San Francisco Bay Area based on the 2018–2023 ACS data as a visual reference (Supplementary Figure S2). Census tract data was not consistently available for percentile of insured adults, percentile of patients with vision difficulty, or percentile of non-English speaking residents. Patient testimonials were shared during clinical follow-up and relayed to the study team via a scleral lens provider; statements were de-identified and included to illustrate patient-perceived impact (Table 2).

**Table 2:** Patient testimonials.

**Testimonial A:**
A female adolescent with a history of bilateral corneal scarring presented with longstanding legal blindness after correction. She reported difficulty in school, inability to obtain employment, and strained familial relationships. She was enrolled in the ACCESS program and underwent fitting with scleral contact lenses. The expected outcome was improved optical correction through ocular surface protection and tear reservoir maintenance. At follow-up, the patient achieved a corrected VA of 20/30 in both eyes. She described renewed academic engagement and reduced distress. Her mother additionally described significant improvement in patient's affect and in family dynamics, telling the ophthalmologist: “Thank you Doctor, for giving me back my daughter.”
**Testimonial B:**
A middle-aged woman presented with a longstanding history of severe dry eye disease refractory to conventional therapies, including punctal plugs, multiple lubricant formulations, and topical cyclosporine (Restasis). She reported debilitating symptoms that required regular use of eye drops every 30 min and keeping her eyes closed to relieve discomfort. She experienced severe functional limitations, often avoiding leaving home or relying on her husband to guide her by hand when walking outdoors. Upon examination, she demonstrated bilateral stage 2 corneal scarring with a corrected VA of 20/80 in both eyes. She was enrolled in the ACCESS program and fitted with scleral contact lenses. The treatment plan involved regular follow-up to support adjusting to lens use and handling. After four months, she achieved proficiency in independent lens insertion and removal, and her corrected VA improved to 20/20 in both eyes. The patient reported significant improvement in ocular comfort and quality of life, transitioning from social withdrawal and emotional distress to active engagement in daily activities. Her husband noted that the couple could now enjoy time outside the home together. At her final follow-up, the patient expressed deep gratitude to her provider, stating: “Doctor, you saved my life.”

The following patient testimonials include unsolicited narrative statements from individuals enrolled in the ACCESS program and were provided to the research team through a contracted scleral lens provider.

Continuous variables were summarized as mean (SD) or median [Interquartile Range (IQR)], as appropriate, and categorical variables as counts and percentages. Snellen visual acuity values were converted to logMAR using the formula $-\log_{10}\left(V\right)$, where V represents Snellen acuity in 20/distance format.^23^ For patients with pre- and post-treatment visual acuity data, logMAR values and visual disability outcomes were compared using a Wilcoxon signed-rank test, a paired t-test, and McNemar's test. All tests were two-sided; p < 0.05 was considered statistically significant. To visually illustrate changes in acuity, Gaussian blur was applied to images of a standard Snellen chart and a high-resolution photograph of the ZSFG hospital entrance to simulate average pre- and post-treatment vision.^24^ All analyses and visualizations were performed using *R* and *Python*.^25^^,^^26^

This study was conducted with approval and in accordance with the UCSF Institutional Review Board (IRB) and San Francisco General Hospital Panel IRB (#24-43270). De-identified analysis was performed retrospectively, and written informed consent was waived by the institutions as the study involved no more than minimal risk to participants.

## Results

**Fig. 2:**
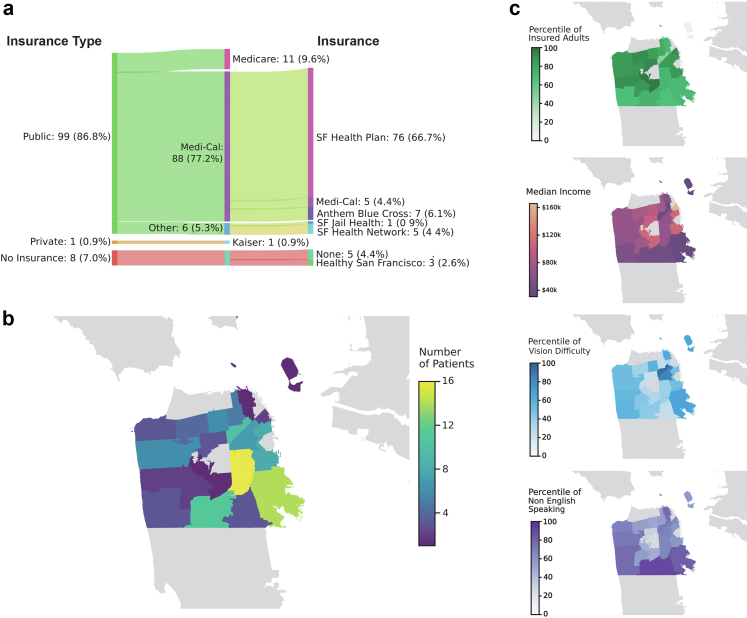
**Patient insurance, residence, and neighborhood context.** (a) Patient insurance type distribution based on broad groups (left) and specific insurance types (right). The number of patients and percentages are shown. (b) Map of San Francisco zip codes colored by the number of ACCESS patients who live in those zip codes. (c) Maps of zip-code-mapped data from American Community Survey (Percentile insured adults, median income) and Healthy Places Indices (Vision difficulty percentile, non-English speaking percentile).

As of June 2025, 114 patients had enrolled in the ACCESS program. Among these patients, the distribution was nearly balanced by sex (50.9% male, 49.1% female), and most patients were under 65 years of age (88.6%) (Table 1). The population predominantly identified as Latine (44.7%), Asian (22.8%), and White (14.9%). The majority of patients had public insurance (105, 92.1%), most commonly through San Francisco's primary Medicaid-managed care plan, SF Health Plan (66, 57.9%), while 8 patients (7.0%) were uninsured (Fig. 2a). Although many lived near ZSFG, particularly in Mission/Bernal Heights (16, 14.0%) and Bayview (14, 12.3%), the program served individuals throughout San Francisco (Fig. 2b). The median [IQR] single-person household income in 2023 for census tracts associated with ACCESS patient residence was $51,383 [$36,796; $75,710] USD (Supplementary Figure S3). According to the California Department of Housing and Community Development, a single-person household income below $104,000 USD in San Francisco during 2023 was considered low income, and income below $65,250 USD was considered “very low income”.^27^ Based on this, over 92% of ACCESS patients lived in neighborhoods where the median income was considered low, and over half in neighborhoods where it was very low. Spatial overlays of participant ZIP-code distribution based on American Community Survey and Healthy Places Index data help visually illustrate overlap with neighborhoods having lower insurance coverage and income, as well as higher proportions of non-English-speaking residents and higher rates of vision difficulty (Fig. 2c).

**Table 1:** Demographic, ocular, and treatment characteristics of patients.

	All patients	Completed treatment
**Number of patients**	114	72
**Number of eyes**	171	121
**Laterality, patient (%)**		
Both eyes	70 (61.4)	49 (68.1)
Left eye	22 (19.3)	9 (12.5)
Right eye	22 (19.3)	14 (19.4)
**Sex, patient (%)**		
Female	56 (49.1)	34 (47.2)
Male	58 (50.9)	38 (52.8)
**Derived race and ethnicity, patient (%)**		
Asian	26 (22.8)	17 (23.6)
Black	10 (8.8)	5 (6.9)
Latine	51 (44.7)	33 (45.8)
Multiple	6 (5.3)	5 (6.9)
NHPI	2 (1.8)	1 (1.4)
Other	2 (1.8)	2 (2.8)
White	17 (14.9)	9 (12.5)
**Insurance type, patient (%)**		
Private	1 (0.9)	1 (1.4)
Public: Medi-Cal	88 (77.2)	58 (80.6)
Public: Medicare	11 (9.6)	4 (5.6)
Public: Other (SF Health Network, SF Jail Health)	6 (5.3)	4 (5.6)
None	8 (7.0)	5 (6.9)
**Primary language, patient (%)**		
Cantonese	6 (5.3)	4 (5.6)
English	67 (58.8)	41 (56.9)
Other	4 (3.5)	3 (4.2)
Russian	3 (2.6)	2 (2.8)
Spanish	34 (29.8)	22 (30.6)
**Age group, patient (%)**		
0–18	13 (11.4)	11 (15.3)
19–39	39 (34.2)	20 (27.8)
40–64	49 (43.0)	36 (50.0)
65+	13 (11.4)	5 (6.9)
**Treatment status, patient (%), eyes (%)**		
Complete	72 (63.2), 121 (70.8)	72 (100.0), 121 (100.0)
Declined	10 (8.8), 13 (7.6)	0 (0.0), 0 (0.0)
Deferred	2 (1.8), 3 (1.8)	0 (0.0), 0 (0.0)
In progress	30 (26.3), 34 (19.9)	0 (0.0), 0 (0.0)
**Diagnosis category, eyes (%)**		
Corneal ectasia	84 (49.1)	58 (47.9)
Corneal opacity	36 (21.1)	25 (20.7)
Dry eye	3 (1.8)	2 (1.7)
Other ocular pathology	14 (8.2)	10 (8.3)
Refractive error of non-corneal origin	34 (19.9)	26 (21.5)
**Treatment, eyes (%)**		
Compounded medication	10 (5.8)	10 (8.3)
Glasses	2 (1.2)	2 (1.7)
None	52 (30.4)	2 (1.7)
RGP lens	33 (19.3)	33 (27.3)
Scleral lens	70 (40.9)	70 (57.9)
Soft contact lens	4 (2.3)	4 (3.3)
**Change in LogMAR, eyes, mean (SD)**	N/A	−0.6 (0.5)

NHPI: Native Hawaiian and Pacific Islander; SF: San Francisco; RGP: rigid gas-permeable; logMAR: logarithm of the Minimum Angle of Resolution; SD: standard deviation.

Characteristics are shown for all enrolled patients (114 patients, 171 eyes) and for those who completed treatment (72 patients, 121 eyes). Variables include laterality, sex, race and ethnicity, insurance type, primary language, age group, treatment status, diagnostic category, and treatment type. Race and ethnicity are reported using the UCSF Health Derived Race and Ethnicity classification, and individuals with documentation consistent with more than one race/ethnicity are classified as “Multiple” and are counted once. Data are presented as n (%), unless otherwise indicated. Percentages are calculated by patients or eyes, as noted. Change in visual acuity is reported as mean (SD) logMAR.

**Fig. 3:**
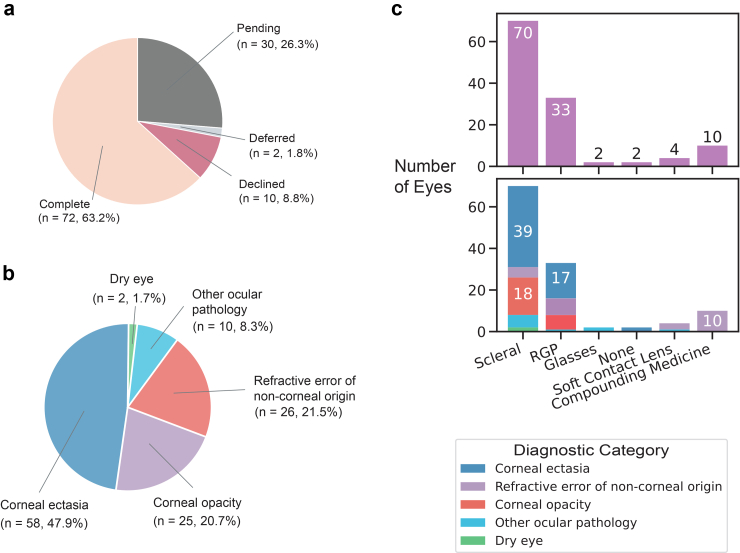
**Diagnoses and treatments.** (a) Breakdown of treatment status for all patients in the ACCESS program. (b) Diagnostic categories for ACCESS referral indications for all eyes with completed treatments. (c) Breakdown of the treatments given to eyes (top) and broken down by diagnostic indications (bottom).

Among all ACCESS patients, 72 (63.2%) completed treatment, accounting for a total of 121 eyes (Fig. 3a, Table 1). Of the 42 (36.8%) patients who have not completed treatment, 30 (26.3%) are pending treatment, 10 (8.8%) declined treatment, and 2 (1.8%) were deferred (Table 1). Among eyes that completed treatment, corneal ectasia was the leading indication (58 eyes, 47.9%), followed by corneal opacity (25, 20.7%) (Fig. 3b, Table 1). Within corneal ectasia, keratoconus was most common (56 eyes, 96.6%), followed by pellucid marginal degeneration (2, 3.4%). For corneal opacity, the primary cause was corneal scarring (17, 70.8%), with other indications including herpes simplex virus (HSV) keratitis (3, 12.5%), limbal stem cell deficiency (3, 12.5%), and corneal ulcer (1, 4.2%) (Table 3). Treatments provided included scleral lenses (70, 57.9% of treated eyes), RGP lenses (33, 27.3%), and compounded eye drop medications (10, 8.3%) (Fig. 3c).

**Table 3:** Distribution of diagnoses among eyes enrolled in the ACCESS program.

Diagnostic category	Indication	N (% of All Eyes)	N (% of All Eyes with Complete Treatment)
Corneal ectasia	Keratoconus	77 (45.0%)	56 (46.3%)
	Pellucid marginal degeneration	5 (2.9%)	2 (1.7%)
	Terrien's marginal degeneration	2 (1.2%)	0 (0.0%)
	**Subtotal**	**84 (49.1%)**	**58 (47.9%)**
Corneal opacity	Band keratopathy	1 (0.6%)	1 (0.8%)
	Corneal scar	22 (12.9%)	17 (14.0%)
	Corneal ulcer	3 (1.8%)	1 (0.8%)
	Corneal ulcer and HSV keratitis	1 (0.6%)	0 (0.0%)
	Granular corneal dystrophy	1 (0.6%)	0 (0.0%)
	HSV keratitis	3 (1.8%)	3 (2.5%)
	Iridocorneal endothelial syndrome	1 (0.6%)	0 (0.0%)
	Limbal stem cell deficiency	4 (2.3%)	3 (2.5%)
	**Subtotal**	**36 (21.1%)**	**25 (20.7%)**
Dry eye	Keratoconjunctivitis sicca	2 (1.2%)	2 (1.7%)
	Superficial punctate keratitis	1 (0.6%)	0 (0.0%)
	**Subtotal**	**3 (1.8%)**	**2 (1.7%)**
Other ocular pathology	Acute retinal necrosis	1 (0.6%)	1 (0.8%)
	Cataract	2 (1.2%)	2 (1.7%)
	Congenital cataract	1 (0.6%)	0 (0.0%)
	Dislocated IOL	1 (0.6%)	1 (0.8%)
	Neurotropic cornea	1 (0.6%)	0 (0.0%)
	Nuclear cataract	4 (2.3%)	4 (3.3%)
	Retinal detachment	2 (1.2%)	1 (0.8%)
	Severe glaucoma, monocular	2 (1.2%)	1 (0.8%)
	**Subtotal**	**14 (8.2%)**	**10 (8.3%)**
Refractive error of non-corneal origin	Anisometropia	4 (2.3%)	3 (2.5%)
	High degree astigmatism	4 (2.3%)	2 (1.7%)
	High myopia	5 (2.9%)	5 (4.1%)
	Irregular astigmatism	2 (1.2%)	2 (1.7%)
	Myopia	8 (4.7%)	8 (6.6%)
	Myopic degeneration	4 (2.3%)	2 (1.7%)
	Ocular albinism, high myopia	1 (0.6%)	0 (0.0%)
	Progressive myopia	2 (1.2%)	0 (0.0%)
	Refractive error	4 (2.3%)	4 (3.3%)
	**Subtotal**	**34 (19.9%)**	**26 (21.5%)**
Total		**171**	**121**

HSV: Herpes simplex virus; IOL: Intraocular lens.

Eyes are grouped by diagnostic category and indication, with counts and percentages shown for all eyes enrolled and all eyes with complete treatment. Diagnostic categories include corneal ectasia, corneal opacity, dry eye, other ocular pathology, and refractive error of non-corneal origin. Percentages are calculated relative to the total number of eyes in each column.

**Fig. 4:**
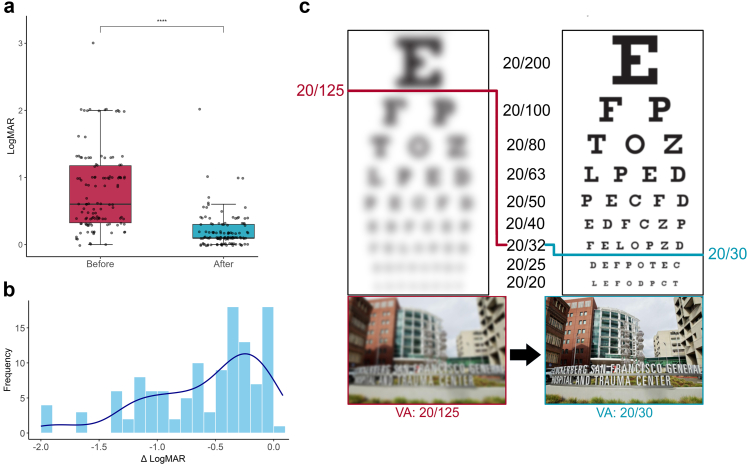
**Improvements in visual acuity.** (a) Boxplot showing visual acuities (LogMAR) for all patients before and after treatment (n = 121 eyes from 72 patients). Visual acuity improved significantly after treatment (p < 0.0001; Wilcoxon signed-rank test), with median logMAR values decreasing from 0.602 [0.325–1.176] to 0.097 [0.097–0.301]. (b) Histogram of change in logMAR values for all treated eyes, with a density curve overlaid (dark blue). (c) Simulated images of Snellen chart showing average visual acuity before (20/125) and after (20/30) treatment. Simulated images of ZSFG hospital entrance at the same levels of average visual acuity. Gaussian blur was applied to approximate visual degradation.

Patients completing treatment had a significant improvement in logMAR visual acuity (p < 0.0001 by two-sided Wilcoxon signed-rank test), with median logMAR values [IQR] improving from 0.602 [0.325–1.176] before treatment to 0.097 [0.097–0.301] after treatment (Fig. 4a, Supplementary Table S2). Most patients achieved a clinically significant improvement in visual acuity (≥0.2 logMAR), with some improving by as much as 2.0 logMAR (Fig. 4b). The mean change ± standard deviation (SD) in logMAR visual acuity was −0.6 ± 0.5, and this corresponded to a mean visual acuity improvement of five Snellen lines (from 20/125 to 20/30, p < 0.0001, paired t-test, Fig. 4c). Of the 72 patients who received treatment, 48 (66.7%) were visually disabled in at least one eye before treatment, defined as visual acuity of 20/200 or worse, compared with only 4 (5.6%) after treatment (p < 0.0001 by McNemar's test).

Estimated costs for ACCESS-covered eye treatments ranged from approximately $550–650 USD per eye for scleral lenses, $105–275 USD per eye for RGP lenses, $90–760 USD per patient for soft contact lenses, $67–251 USD for a one-month supply of compounded eye drop medications, and $100–200 USD for glasses (Table 4, Supplementary Table S3). Additionally, typical costs for initial fittings ranged from $688 to 810 for sclerals USD, $136–210 USD for RGPs, and $119–210 USD for soft contact lenses (Table 4). Applying these estimates to treatments delivered through ACCESS yields a projected annual cost savings to patients of roughly $79,000–112,000 USD, comprising 70 eyes treated with scleral lenses, 33 eyes with RGP lenses, 2 patients with soft contact lenses, 5 patients with compounded medications, and one patient with glasses (Table 1). Using the estimate by Rein et al. of $16,383 USD in annual economic cost per person with visual disability, the 48 patients who were visually disabled before treatment represented an estimated annual economic impact of $808,224 USD.^7^ Restoration of functional vision in 44 of these patients corresponds to an estimated annual reduction in economic impact of $720,852 USD.

**Table 4:** Estimated costs of treatments covered by the ACCESS program.

	Number of patients treated	Number of eyes treated	Typical cost per intervention ($ USD)	Typical cost per initial fitting ($ USD)	Estimated total cost ($ USD)^d^
Scleral lenses^a^	44	70	550–650 per eye	688–810	68,772–81,140
RGP lenses^a^	20	33	105–275 per eye	136–210	6185–13,275
Soft contact lenses^a^	2	4	90–760 per patient	119–210	299–1940
Compounded medication^b^	5	10	804–3012 per patient	–	4020–15,060
Glasses^c^	1	2	100–200 per patient	–	100–200
Total	72	119^e^	–	–	**79,376**–**111,615**

Data show the number of patients and eyes treated, estimated average cost per eye, and total projected cost for three treatment types supported by ACCESS. These interventions were provided at no cost to patients with limited or no insurance coverage. The total reflects the estimated financial burden alleviated by the program.

a Pricing for scleral lenses, RGP lenses, soft contact lenses, and lens fittings based on UCSF Health charges, with estimated treatment for a year.

b Pricing for a one-year supply of compounded eye drop medications for bilateral use in 2022 from accredited compounding pharmacies contracted with the ACCESS program, estimated by extrapolating monthly supply costs over 12 months (Supplementary Table S3).

c Pricing for glasses based on the range of out-of-pocket costs for patients insured by SF Health Plan.

d For per-eye interventions (scleral, RGP), estimated total costs were calculated as: (Number of eyes × typical cost per intervention) + (Number of patients × typical cost per initial fitting). For per-patient interventions (soft contact lenses, compounded medications, glasses), estimated total costs were calculated as: (Number of patients × typical cost per patient).

e Two eyes from one patient who declined treatment are excluded from total.

Beyond quantitative measures of cost savings and reductions in disability burden, the ACCESS program also yielded profound qualitative improvements in patients’ lived experiences (Table 2). These representative patient testimonials underscore how vision-restorative care not only improves functional vision, but also translates to personal independence, social reintegration, familial connection, academic participation, and emotional well-being, thereby amplifying the clinical and economic value of such programs.

## Discussion

ACCESS demonstrates that modest, well-targeted investments can produce exponential clinical and societal benefits. By filling critical limitations in insurance coverage at a public safety-net hospital, the program restored functional vision for the majority of participants—transforming not only their vision but their independence, productivity, and capacity to engage with families, education, and work. These findings show that relatively low-cost, high-yield interventions can reduce preventable disability and serve as a replicable model for advancing equity in specialty vision care, while simultaneously providing downstream cost savings through the reduction of visual disability.

ACCESS has focused on high-impact treatments that do not have viable alternatives. Scleral and RGP lenses can restore vision in patients with irregular corneas that are untreatable with glasses or soft contact lenses, while compounded medications can be the best or only viable therapies for certain ocular diseases, such as severe infectious keratitis and ocular surface neoplasia. Yet these interventions are often excluded or undercovered by insurance, leaving many of the highest-need patients without appropriate care.

The distribution of diagnoses in this cohort and the program's approach to treatment delivery reflect this focus. As shown in Table 3, nearly half of all examined eyes from enrolled patients had corneal ectasia, most often attributed to keratoconus; about one-fifth had corneal opacity, most often due to corneal scarring; and another fifth had refractive error of non-corneal origin, including high myopia and irregular astigmatism. These are precisely the conditions for which scleral and RGP lenses are uniquely capable of regularizing the ocular surface and restoring functional visual acuity. Other diagnostic groups highlight the program's current scope, including severe dry eyes and other ocular pathologies, with some cases requiring compounded medications. These medications are frequently excluded from insurance coverage and often difficult to obtain in the outpatient setting. By partnering with accredited compounding pharmacies to ship medications directly to patients, ACCESS helps overcome in-house dispensing limitations and reduces geographic and cost barriers to timely medication retrieval.

### Economic Implications

Among patients who completed therapy in the ACCESS program, 30 were treated for keratoconus, making this the most common diagnosis among enrollees. Given a keratoconus prevalence of 1 in 2500 in the United States and San Francisco's estimated population of 813,000–828,000 between 2021 and 2024, approximately 325–330 individuals in the city are expected to be living with keratoconus at any given time.^28^^,^^29^ As ZSFG handles over 20 percent of the city's inpatient care and serves over 100,000 patients annually, the 30 patients treated through ACCESS likely constitute a significant share of the keratoconus cases seen at this hospital.^8^ Still, inequities in vision health remain pronounced. Corneal ectasias are disproportionately borne by under-resourced populations, with an estimated lifetime cost of more than $43,000 USD.^30^ Prior studies have shown that non-English-speaking patients experience lower rates of keratoconus treatment and longer hospitalizations for corneal ulcers, while non-white ethnicity, lack of private insurance, and low household income are strongly associated with uncorrected refractive errors.^31^^,^^32^ The demographics of our patient population—predominantly publicly insured individuals, living in neighborhoods with low median income, high proportions of non-English-speaking residents, and elevated rates of vision difficulty—reflect that ACCESS effectively reached those at greatest risk. Nevertheless, future initiatives should focus on strengthening screening, diagnosis, and referral pathways for keratoconus and other progressive corneal diseases, particularly within public health systems that serve high-risk patient populations.

The impact of restoring vision extends well beyond clinical measures. Patients who regain functional sight are more likely to re-enter the workforce, reduce dependence on disability support programs, and contribute productively to their families and communities. Among patients treated through ACCESS, restoration of functional vision in 44 patients corresponded to an estimated reduction of more than $720,000 USD in annual economic impact. The true societal value is likely far greater, encompassing enhanced mental health, family reintegration, and reduced caregiver strain. Moreover, the program yields important system-level benefits. At ZSFG, compounded eye drops cannot be dispensed on a continuing outpatient basis, often necessitating inpatient admission for conditions such as severe infectious keratitis. By enabling ongoing outpatient access to these medications through a partner pharmacy, ACCESS helps prevent avoidable hospitalizations for drop administration and decreases the need for alternate surgical treatments. With an average hospital stay of approximately 7 days for infectious keratitis and an inpatient expense of $4471 USD per day in California, the estimated cost savings amount to roughly $31,000 USD per patient.^31^^,^^33^ Furthermore, the per-patient cost of treatments provided through ACCESS was markedly lower. Scleral lenses represented the most significant expenditure, with an estimated program cost between $68,722–81,140 USD for 44 patients (70 eyes); RGP lenses were the second largest at $6185–13,275 USD for 20 patients (33 eyes); and compounded medications totaled $4020–15,060 USD for 5 patients (Table 4). Smaller but meaningful contributions included soft contact lenses and glasses, which accounted for $299–1940 USD for 2 patients and $100–200 USD for 1 patient, respectively (Table 4). In total, the program's estimated outlay of $79,376–111,615 USD supported 72 patients, nearly all of whom experienced substantial improvements in visual acuity. When considered alongside the projected $720,000 USD reduction in annual economic impact from restoration of functional vision, these findings demonstrate a favorable cost–benefit profile and suggest a high potential return on investment for insurers and health systems. This evidence provides a foundation to help guide hospital policy development on cost-coverage programs and supports broader expansion of insurance coverage for essential vision-restorative treatments.

### Challenges and limitations

Despite its early success, ACCESS must overcome challenges to ensure sustainability. Continued funding depends on consistent data collection and demonstrable outcomes, which in turn require protocolized workflows and durable infrastructure. Reliance on medical students and rotating volunteers makes the program vulnerable to lapses in continuity, underscoring the need for systematic documentation and structured leadership transitions. Yet this model also provides formative training opportunities in equity-driven care. Engaging students in service-learning experiences has been shown to cultivate empathy, leadership, and social accountability, while fostering long-term commitment to health equity.^29^

Beyond these operational considerations, the study has several methodological limitations. As a single-site program at a safety-net hospital, findings may not be fully generalizable to other populations or healthcare systems. The sample size remains modest, and treatment completion at the time of analysis was influenced by practical barriers such as scheduling delays and loss to follow-up. Visual acuity outcomes were not available for all treated patients, and among those with available data, the timing of measurements varied—most obtained on the day of lens fitting, but some months later—introducing heterogeneity in outcome assessment. Standardized measures of quality of life, employment status, and functional independence were not systematically collected, limiting the ability to fully capture the broader impact of ACCESS interventions, while the absence of a control group prevents causal inference. In particular, we were unable to compare treatment completion rates to a prior institutional baseline because ACCESS established the first prospective, programmatic registry for these noncovered interventions. Before implementation, specialty lenses and compounded therapies were obtained through fragmented and often external pathways (e.g., multiple vendors and ad hoc charitable mechanisms) that were not captured in a standardized, trackable workflow with discrete documentation fields. Future iterations should incorporate longitudinal monitoring of treatment completion rates and related implementation metrics to better contextualize program impact over time. Accordingly, this evaluation was not designed as a randomized clinical trial, but rather as a pragmatic cohort study grounded in implementation science, aimed at examining real-world care delivery in under-resourced settings and at examining how systemic barriers can be overcome to meet community needs.

### Existing efforts

Several national and institutional initiatives in ophthalmic care, as well as global commission reports on eye health, have informed the development of ACCESS and provide helpful context on the societal value of targeted, high-yield ocular interventions. EyeCare America is a public service program of the American Academy of Ophthalmology that connects eligible patients with volunteer ophthalmologists for a medical eye exam,^34^ while AGS CARES provides surgical glaucoma care through a network of volunteer glaucoma specialists.^35^ These models are sustained largely through philanthropy and foundation support combined with donated professional time and services. Similarly, many academic centers offer reduced-fee or subsidized eye care services, though these programs often focus on basic refractive or medical management. Community outreach and mobile screening initiatives have further improved disease detection and referral pathways for underserved populations. For example, Bascom Palmer Eye Institute's Vision Van and the UCLA Mobile Eye Clinic—both supported by charitable funding—deliver free community vision screenings and facilitate referral to ophthalmic care, collectively reaching thousands of individuals locally and globally.^36^ Collectively, these efforts demonstrate the feasibility and public health impact of coordinated, philanthropy-supported models that bridge coverage gaps in vision care.

Notably, despite the existence of philanthropic, volunteer, and voucher-based initiatives, there are few peer-reviewed evaluations of hospital-embedded cost-coverage programs for specialty ophthalmic care in US safety-net settings. ACCESS was developed in the spirit of these longstanding efforts and extend their reach by identifying and delivering high-yield specialty therapies to patients lacking adequate insurance coverage. In doing so, ACCESS directly tackles challenges in vision health equity emphasized and prioritized by global eye health commission reports: reaching vulnerable, underinsured, linguistically diverse patients; responding to unmet needs in underserved communities with cost-effective interventions; and eliminating out-of-pocket costs for essential vision-restorative care.^15^^,^^16^

Importantly, the ACCESS model is adaptable beyond ZSFG. In the United States, approximately 25% of hospitals qualify as safety-net institutions, defined as medical centers with the highest proportion of Medicaid and uninsured discharges.^30^ By demonstrating proof of concept at one of California's largest public safety-net hospitals, ACCESS illustrates how similar institutions could adopt this model to reduce disparities in specialty care. Since its inception, ACCESS has secured over $700,000 USD in support, including approximately $250,000 USD of initial funding in 2021, and coordinated with local stakeholder groups committed to improving community health. The results presented here are derived primarily from this initial investment. Equally critical, partnership with the All May See Foundation has provided the infrastructure, continuity, and visibility necessary to embed ACCESS within a broader network of institutional support. Building on this foundation, new partnerships with city and county health systems, community-based groups, and nonprofit organizations are now being pursued to further extend the program's reach and sustainability.

In parallel with these partnership-based efforts for long-term sustainability, there is ongoing policy activity aimed at expanding publicly supported vision services, though coverage remains inconsistent and often limited in scope. For example, in California, Medi-Cal includes established pathways for medically necessary contact lenses limited to specific conditions such as keratoconus and corneal pathology or deformity.^37^^,^^38^ Additionally, SB 502 (Chapter 487, 2023) has enabled support for mobile, school-based vision services for low-income children.^39^ DHCS has also launched the Mobile Optometric Services (MOS) Program to provide no-cost vision screening, exams, and glasses in school settings.^40^

At the federal level, proposals have also been introduced to expand Medicare coverage to include vision services. For example, H.R. 2045 (the “Medicare Dental, Vision, and Hearing Benefit Act of 2025”) would add coverage for routine eye exams and corrective lenses under Medicare, but it has been introduced and referred to committee without enactment to date, and its ultimate scope and implementation details (including cost-sharing and program limitations) remain uncertain.^41^ Together, these state and federal efforts underscore growing recognition of the importance of vision coverage, while also highlighting persistent gaps and administrative barriers that can leave patients insufficiently covered for high-impact specialty interventions. In this context, pragmatic, outcomes-driven evidence can help inform policy decisions about which services merit expansion and how to structure sustainable coverage models for essential vision-restorative care.

ACCESS demonstrates significantly improved vision outcomes and reduced financial burden for underinsured patients. Its success illustrates how strategically targeted interventions in safety-net settings can achieve disproportionate gains in health outcomes with relatively modest resources. By aligning equity, efficiency, and sustainability, the program offers a scalable model for expanding access to specialty care and reducing disparities in vulnerable populations.

## Contributors

RAM, IJP, MY, and TNK conceptualized this study. DC, HKK and TNK acquired funding. RAM and IJP wrote the initial draft. RAM, IJP, AST, DC, HKK, MY, and TNK reviewed and edited the manuscript. RAM, IJP, and AST performed data curation. RAM, IJP and AST conducted the statistical analysis. RAM, IJP, AST, MY, and TNK performed investigation and methodology. MY and TNK supervised this study. RAM, IJP, AST, MY, and TNK had full access to and verified the underlying data and data analysis. All authors contributed to data interpretation, revised the manuscript for important intellectual content, and agreed to submit the manuscript for publication. RAM, IJP, and AST contributed equally to this work.

## Data sharing statement

De-identified participant data that support the findings of this study will be available from the corresponding author following publication upon reasonable request, contingent on institutional approval and compliance with patient confidentiality and ethical regulations. Analysis code will also be made available to enable replication by investigators with authorized access to similar data. Please email Tyson Kim at tyson.kim@ucsf.edu to request a copy of the code.

## Editor note

The Lancet Group takes a neutral position with respect to territorial claims in published maps and institutional affiliations.

## Declaration of interests

The authors declare no competing interests.
