# Supplementary material for: Alleviating Costs for Critical Eye Specialty Services (ACCESS): a prospective cohort analysis of a cost-coverage program at a public safety-net hospital
Source: Lancet Reg Health Am. 2026 Mar 13;57:101450. doi: 10.1016/j.lana.2026.101450 (PMC12999287; doi:10.1016/j.lana.2026.101450)
Supplement: Supplementary Material [file mmc1.pdf]

Supplementary Materials

Alleviating Costs for Critical Eye Specialty Services (ACCESS): A Prospective Cohort Analysis of a Cost-Coverage Program at a Public Safety-Net Hospital

Ryan A Morton[1,*], BA, Irene J Pak[1,*], BS, Alice S Tang[1,*], PhD, Deborah Chesky[2], CFRE, Hemal K Kanzaria[3], MD, Madeline Yung[1], MD, Tyson N Kim[1], MD PhD

Affiliations:

1. Department of Ophthalmology, University of California-San Francisco, San Francisco, CA, USA
2. All May See Foundation, San Francisco, CA, USA
3. Department of Emergency Medicine, University of California-San Francisco, San Francisco, CA, USA

* These authors contributed equally to this work.

Table of Contents

Supplementary Table 1: Classification of ocular diagnoses grouped in five categories 2
Supplementary Table 2: Statistics Summary 3
Supplementary Table 3: Medications available to ACCESS patients through compounding pharmacy 4
Supplementary Figure 1: STROBE Diagram 5
Supplementary Figure 2: Median household income by census tract in the San Francisco Bay Area 6
Supplementary Figure 3: Distribution of census-tract median household income for ACCESS participants 7

Supplementary Table 1: Classification of ocular diagnoses grouped in five categories

Diagnostic Category	Specific Diagnoses
**Corneal ectasia**	Keratoconus, Marginal degeneration
**Corneal opacity**	Corneal opacity, Corneal scar, Corneal ulcer, Limbal stem cell deficiency, Granular corneal dystrophy, Iridocorneal endothelial syndrome, Corneal graft, HSV Keratitis, Band keratopathy
**Dry eye**	Superficial punctate keratitis, Keratoconjunctivitis sicca
**Other ocular pathology**	Retinal detachment, Acute retinal necrosis, Severe glaucoma, Congenital cataract, Nuclear cataract, Dislocated intraocular lens, Neurotropic cornea
**Refractive error of non-corneal origin**	Myopia, Refractive Error, Myopia control with atropine, Irregular astigmatism, Anisometropia, Myopic degeneration

Supplementary Table 2: Statistics Summary

	Before treatment	After treatment	Change (after – before)	P-value
**logMAR VA, median [IQR]**	0·602 [0·325–1·176]	0·097 [0·097–0·301]	-0·466 [-0·903– -0·204]	<0·0001*
**logMAR VA, mean ± SD**	0·805 ± 0·592	0·199 ± 0·265	-0·605 ± 0.498	<0·0001†
**Proportion of patients visually disabled ≥ 1 eye, n (%)**	48 (66·7)	4 (5·6)	-44 (-61·1)	<0·0001‡

Statistical analyses were performed on the 72 patients that completed treatment. A Wilcoxon signed-rank test and paired t-test were used to evaluate the median and mean changes in VA before and after treatment.

*Two-sided Wilcoxon signed-rank test

†Two-sided paired t-test

‡Two-sided McNemar's test

Supplementary Table 3: Medications available to ACCESS patients through compounding pharmacy

Compounded Medication	Volume (mL)	Cost ($ USD)*
Atropine (0·01-0·25%)	10	67·95–78·58
Chlorhexidine (0·02%)	15	101·34
Cyclosporin (0·5%)	10	130·41
Dexamethasone (0·1%)	10	113·42
Fluorouracil (1%)	5	98·55
Mitomycin (0·02%)	5	248·24
Polyhexamethylene biguanide (0·02%)	15	94·89
Serum tears (30-50%)	60	250·95
Tacrolimus (0·4%)	5	123·05
Tobramycin (15mg/mL)	10	95·62
Vancomycin (25-50 mg/mL)	10	90·19–99·29

* Pricing for 1 month supply of compounded eye drop medications as of 2022 from accredited compounding pharmacy contracted with ACCESS program.

Supplementary Figure 1: STROBE Diagram

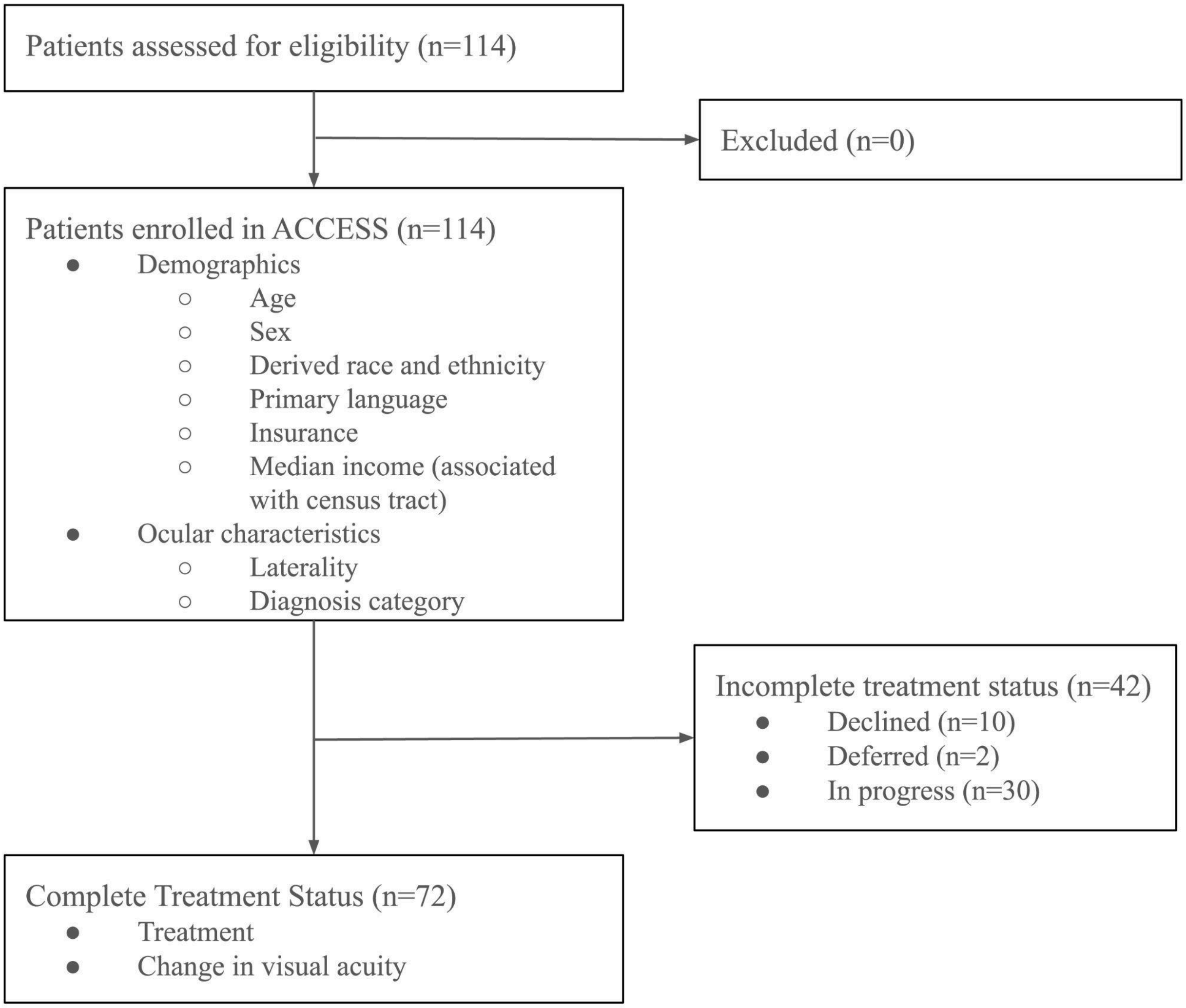

STROBE (Strengthening the Reporting of Observational Studies in Epidemiology) diagram of ACCESS cohort and data availability.

Supplementary Figure 2: Median household income by census tract in the San Francisco Bay Area

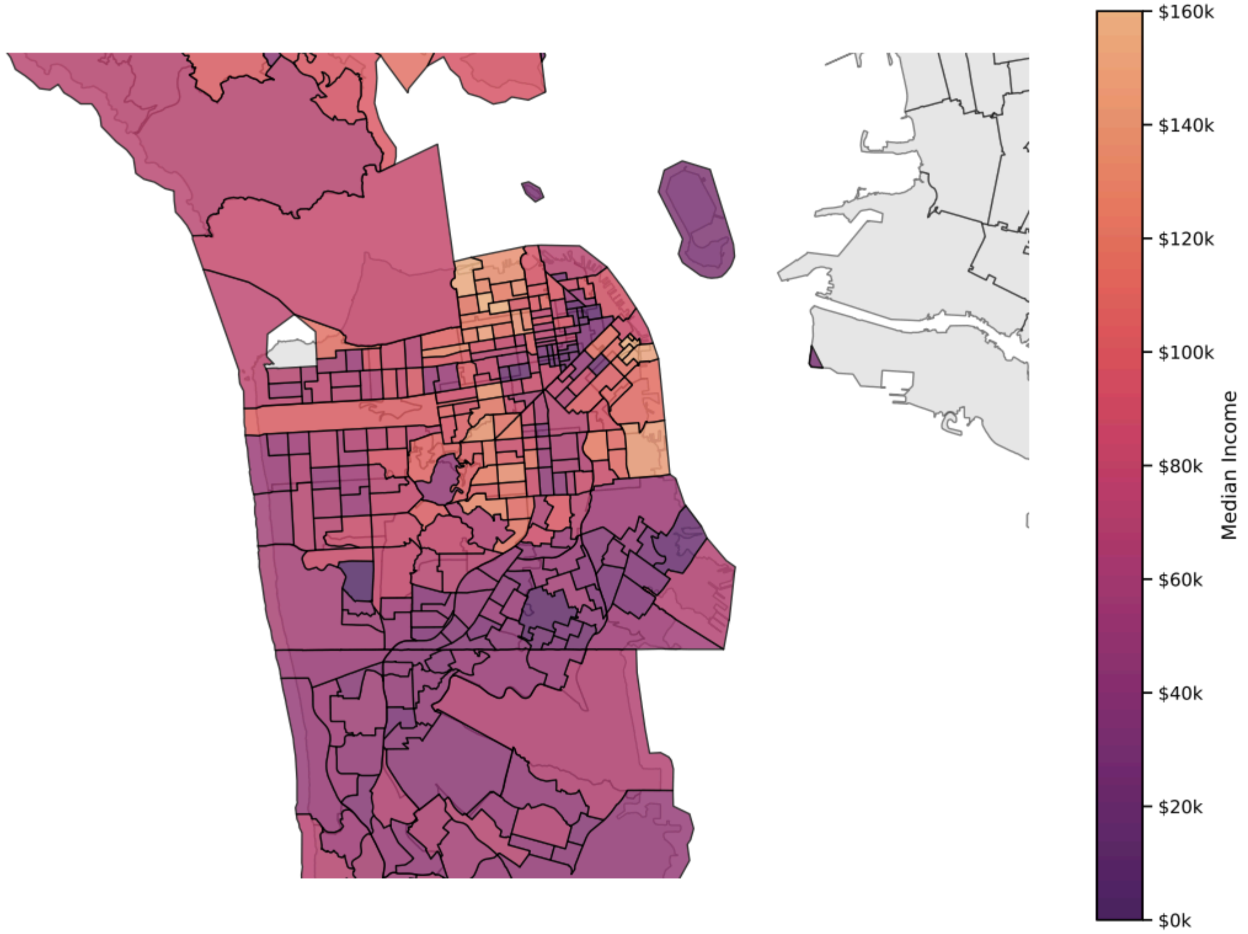

Choropleth map of census-tract median household income (USD) based on American Community Survey (ACS) data from 2018-2023.

Supplementary Figure 3: Distribution of census-tract median household income for ACCESS participants

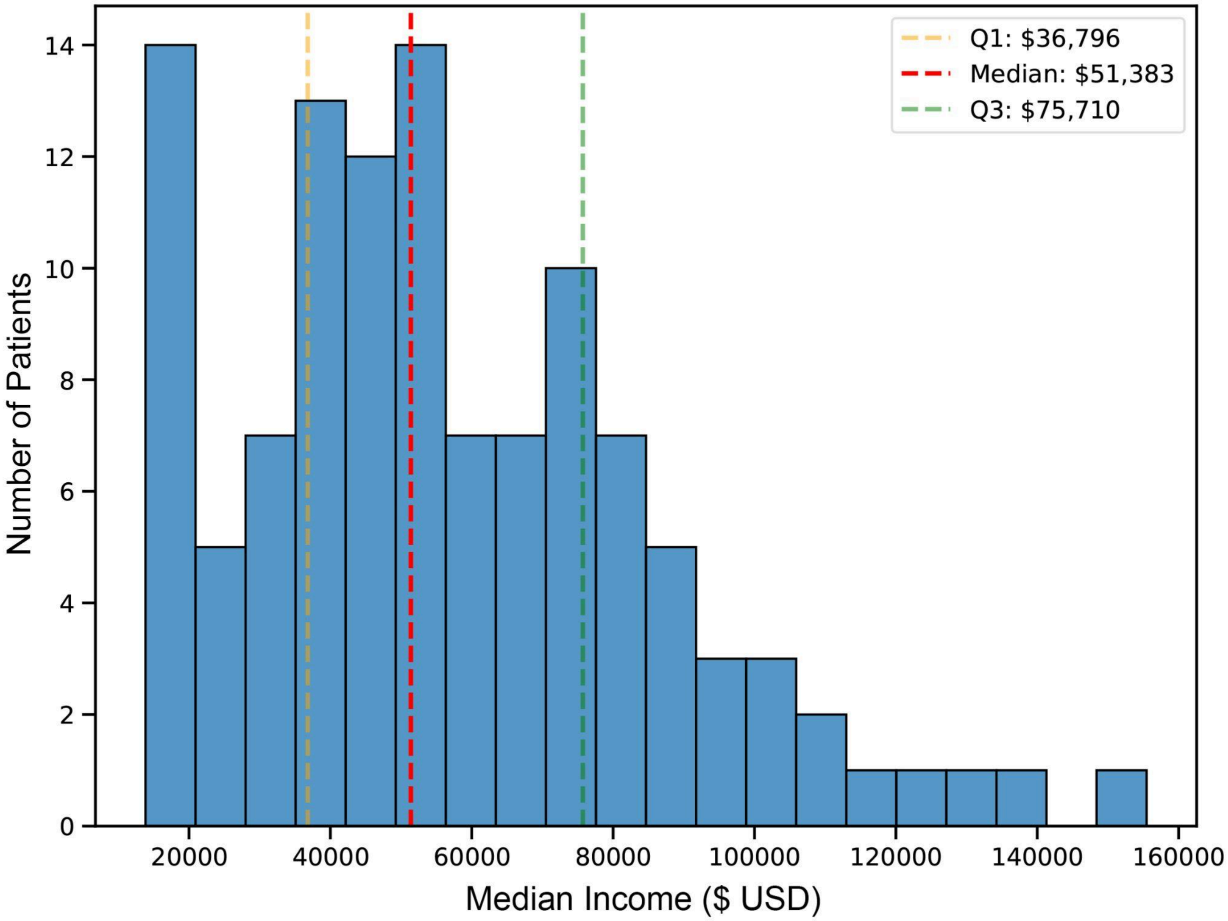

Histogram of American Community Survey–derived median single-person household income (USD) for census tracts corresponding to the residential locations of all 114 patients enrolled in ACCESS. Patients were binned into 20 equal-width income intervals spanning $13,846–$155,468. Vertical markers indicate the sample quartiles (Q1: $36,796; median: $51,383; Q3: $75,710).
